# Phantom Acupuncture Induces Placebo Credibility and Vicarious Sensations: A Parallel fMRI Study of Low Back Pain Patients

**DOI:** 10.1038/s41598-017-18870-1

**Published:** 2018-01-17

**Authors:** Meena M. Makary, Jeungchan Lee, Eunyoung Lee, Seulgi Eun, Jieun Kim, Geon-Ho Jahng, Kiok Kim, You-Suk Youn, Jun-Hwan Lee, Kyungmo Park

**Affiliations:** 1 Systems and Biomedical Engineering Department, Faculty of Engineering, Cairo University, Giza, 12613 Egypt; 2 Department of Biomedical Engineering, Kyung Hee University, Yongin, 17104 Republic of Korea; 3 Department of Psychiatry, Yale University School of Medicine, New Haven, 06511 CT USA; 4 The John B. Pierce Laboratory, New Haven, 06519 CT USA; 5 Martinos Center for Biomedical Imaging, Department of Radiology, Massachusetts General Hospital, Harvard Medical School, Boston, 02129 MA USA; 6 Clinical Research Division, Korea Institute of Oriental Medicine, Daejeon, 34054 Republic of Korea; 7 Department of Radiology, Kyung Hee University Hospital at Gangdong, College of Medicine, Kyung Hee University, Seoul, 05278 Republic of Korea; 8 Department of Spine Center, Mokhuri Neck & Back Hospital, Seoul, 06272 Republic of Korea; 9 Korean Medicine Life Science, University of Science & Technology (UST), Campus of Korea Institute of Oriental Medicine, Daejeon, 34054 Republic of Korea

## Abstract

Although acupuncture is an effective therapeutic intervention for pain reduction, the exact difference between real and sham acupuncture has not been clearly understood because a somatosensory tactile component is commonly included in the existing sham acupuncture protocols. In an event-related fMRI experiment, we implemented a novel form of sham acupuncture, *phantom acupuncture*, that reproduces the acupuncture needling procedure without somatosensory tactile stimulation while maintaining the credibility of the acupuncture treatment context. Fifty-six non-specific low back pain patients received either real (REAL) or phantom (PHNT) acupuncture stimulation in a parallel group study. The REAL group exhibited greater activation in the posterior insula and anterior cingulate cortex, reflecting the needling-specific components of acupuncture. We demonstrated that PHNT could be delivered credibly. Interestingly, the PHNT-credible group exhibited bilateral activation in SI/SII and also reported vicarious acupuncture sensations without needling stimulation. The PHNT group showed greater activation in the bilateral dorsolateral/ventrolateral prefrontal cortex (dlPFC/vlPFC). Moreover, the PHNT group exhibited significant pain reduction, with a significant correlation between the subjective fMRI signal in the right dlPFC/vlPFC and a score assessing belief in acupuncture effectiveness. These results support an expectation-related placebo analgesic effect on subjective pain intensity ratings, possibly mediated by right prefrontal cortex activity.

## Introduction

Even though acupuncture has been proved effective for pain reduction in many animal studies and clinical trials, the difference between sham and real acupuncture has not been clearly understood^1–3^, in part because a somatosensory tactile component is commonly included in the sham controls. Thus, to better determine the specific effects of acupuncture, it is essential to separate this complex therapeutic intervention into its constituent components. Acupuncture is a multi-dimensional therapeutic treatment; therefore it is helpful to use sham acupuncture methods as controls in clinical trials to better investigate the different dimensions of acupuncture as a therapeutic intervention^4^. Previously used sham acupuncture techniques have included a tactile component to enhance the credibility of the needling ritual, which has made it difficult to understand and separate acupuncture components. Tactile stimulation, which produces physiological and brain responses, could overlap with real acupuncture. Therefore, it would be beneficial to develop a credible sham acupuncture technique that completely excludes tactile (i.e., somatosensory) stimulation^5^. Generally speaking, the acupuncture ritual consists of three different components: needling/tactile somatosensory-specific stimulation, a cognitive component of needling credibility, and an attentional shift component resulting from visual/somatosensory stimulation^6^. In a recent fMRI study, Jung *et al*. aimed to differentiate the brain responses to bodily attention of acupuncture stimulation in case of with and without stimulation. They concluded that, regardless of stimulation type, bodily attention activates the salience network and deactivate the default mode network^7^. In our previous study, we developed a novel form of sham acupuncture, a visual manipulation we dubbed phantom acupuncture, which reproduces the acupuncture needling ritual without somatosensory tactile stimulation^6^. We used psychophysiological and psychophysical measures to dissociate the different acupuncture components, and we found that the needling tactile-specific component induces sympathetic activation, whereas acupuncture contextual effects (needling credibility) result in decreased heart rate (a shift toward cardiovagal activation) during acupuncture needling and decreased pupil size (parasympathetic activity), as well as decreased skin conductance response (sympathetic inhibition) after acupuncture needling. In this study, we used the same phantom acupuncture procedure in a functional MRI (fMRI) experimental design to dissociate the neural correlates of somatosensory and contextual acupuncture components (needling-specific and non-specific) in low back pain patients. In conjunction with fMRI data acquisition, we measured concurrent peripheral autonomic nervous system (ANS) recordings (skin conductance response, SCR; heart rate, HR), similar to our previous study^8^. Combined fMRI-ANS experimental paradigm gives an opportunity to examine the neural correlates of psychophysiological response induced by acupuncture stimulation.

A growing body of evidence supports the existence of mirror systems (mirror neurons that activate during the observation and execution of actions) in both monkey and human brains (see^9^ for review). Moreover, other brain systems have been described with mirror properties. For instance, overlapped activations were reported by the observation and experience of pain^10^, disgust^11^, emotional facial expressions^12^, and touch^13,14^. In the past few decades, several studies have reported the existence of a neural system that contains overlapping activations for real and observed sensations^14–17^. Activation of the somatosensory system while observing touch stimulation is called sensory referral, and several authors have argued that sensory referral might be unconscious, and sometimes it might even lead to conscious sensations of touch quale^13,16,18^.

The experience of pain can be modulated by expectations and beliefs, which is particularly evident in placebo analgesia^19,20^. Growing evidence from neuroimaging^21,22^ and lesion^23^ studies suggests prefrontal cortex involvement in placebo analgesia. Furthermore, prefrontal neural mechanisms are known to mediate placebo analgesia through opioid release in subcortical structures (e.g., midbrain), which subsequently leads to reduction of pain transmission^19,23,24^. In particular, the dorsolateral and ventrolateral prefrontal cortex (dlPFC/vlPFC) have repeatedly been reported to be involved in expectation-related placebo analgesia^19,25–27^ and also in attention-related and cognitive pain regulation^28–33^.

In this study, we used our previously developed phantom acupuncture procedure, which completely excludes somatosensory stimulation while maintaining needling credibility, with an event-related fMRI experimental design in low back pain patients. We thereby aimed to dissociate brain responses to somatosensory needling (needling specific) from the needling credibility and visual stimulation (needling non-specific) components of acupuncture. Furthermore, we hypothesized that phantom acupuncture (when credible) could induce expectation-related placebo analgesic effects mediated by prefrontal cortex activation.

## Methods

### Participants

**Figure 1:**
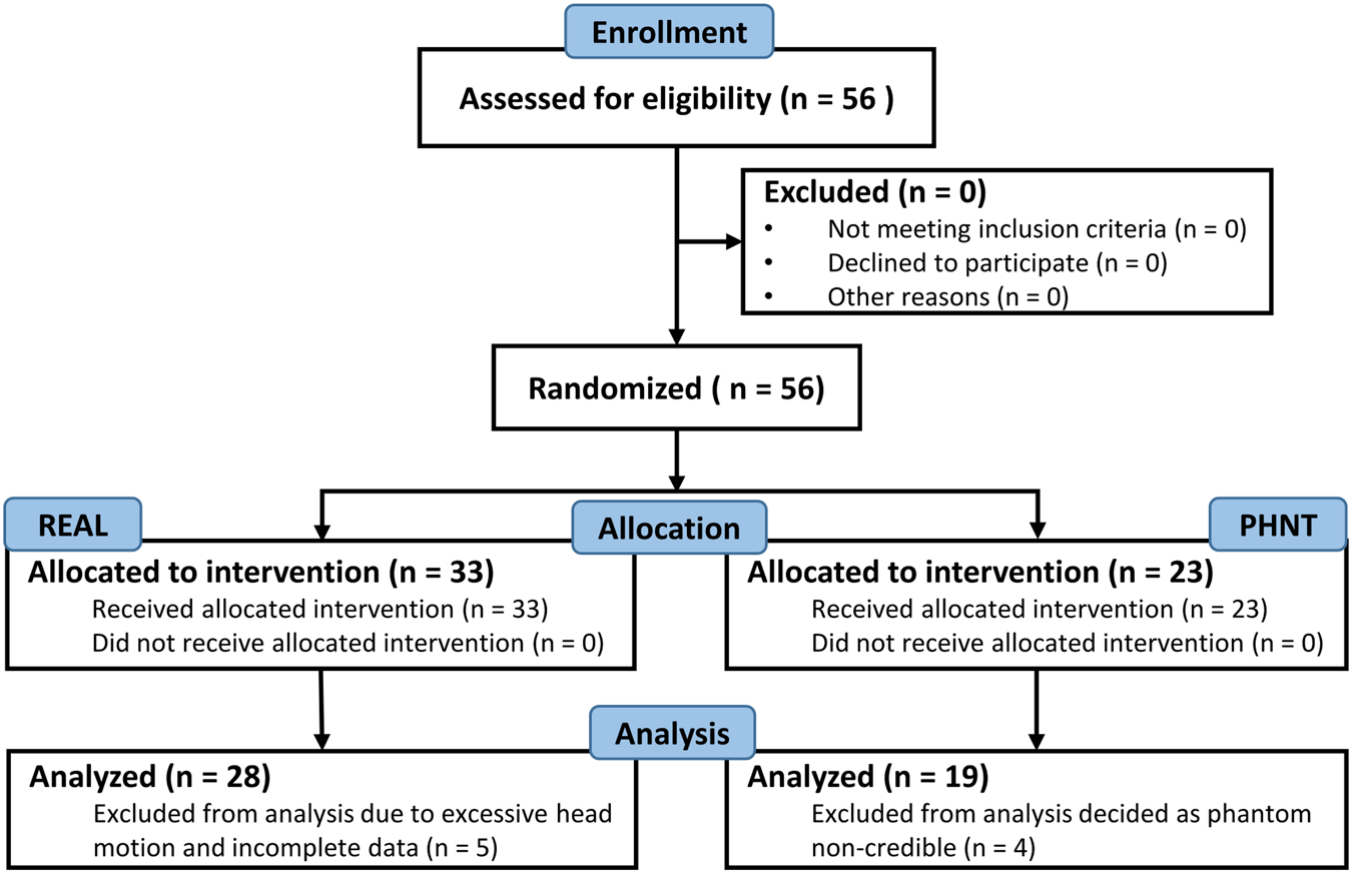
Study flow. Participants flow through the study.

A cohort of 56 non-specific low back pain (LBP) patients (right-handed^34^, 25 females, age = 38.4 ± 12.7 years old, mean ± STD) were enrolled in this study (Fig. 1). We recruited patients from three clinics: Kyung Hee University Hospital at Gangdong, Mokhuri Oriental Medicine Hospital, and Sejongno Medical Clinic, adhering to Kyung Hee University guidelines for recruitment at neighboring hospitals and academic institutions. All participants gave their written informed consent before participating in the experiment, fulfilling the requirements of the Institutional Review Board (IRB) of Kyung Hee University (IRB approval number: KHNMC-OH-IRB 2010-013) and in accordance with the Helsinki Declaration. This trial is publically registered at clinical research information service (CRIS, https://cris.nih.go.kr/, registration number: KCT0002253 and registration date: 2017.03.07). Participants were asked not to take any medications/treatment (e.g., autonomic modulating or pharmacological substances) that might affect this study on the scanning day. We excluded participants from this study if they: (1) were below 19 years old, (2) had severe pain other than LBP (such as neck pain or headache), (3) had severe radicular pain (pain extended to their lower leg), (4) had neurological or cardiovascular disease, (5) had LBP from an external injury (accident or surgery), or (6) had any possible psychological motivation (compensating accident insurance) that might affect the pain rating. Patients who reported evoked pain greater than 4 on a 0–10 visual analog scale (VAS) (0: no pain, 10: maximum imaginable pain) when lifting their low back to a height of 4–7 cm in a supine position were included. In addition, patients were screened to confirm their compatibility and safety in an MRI environment.

### Experimental Design

**Figure 2:**
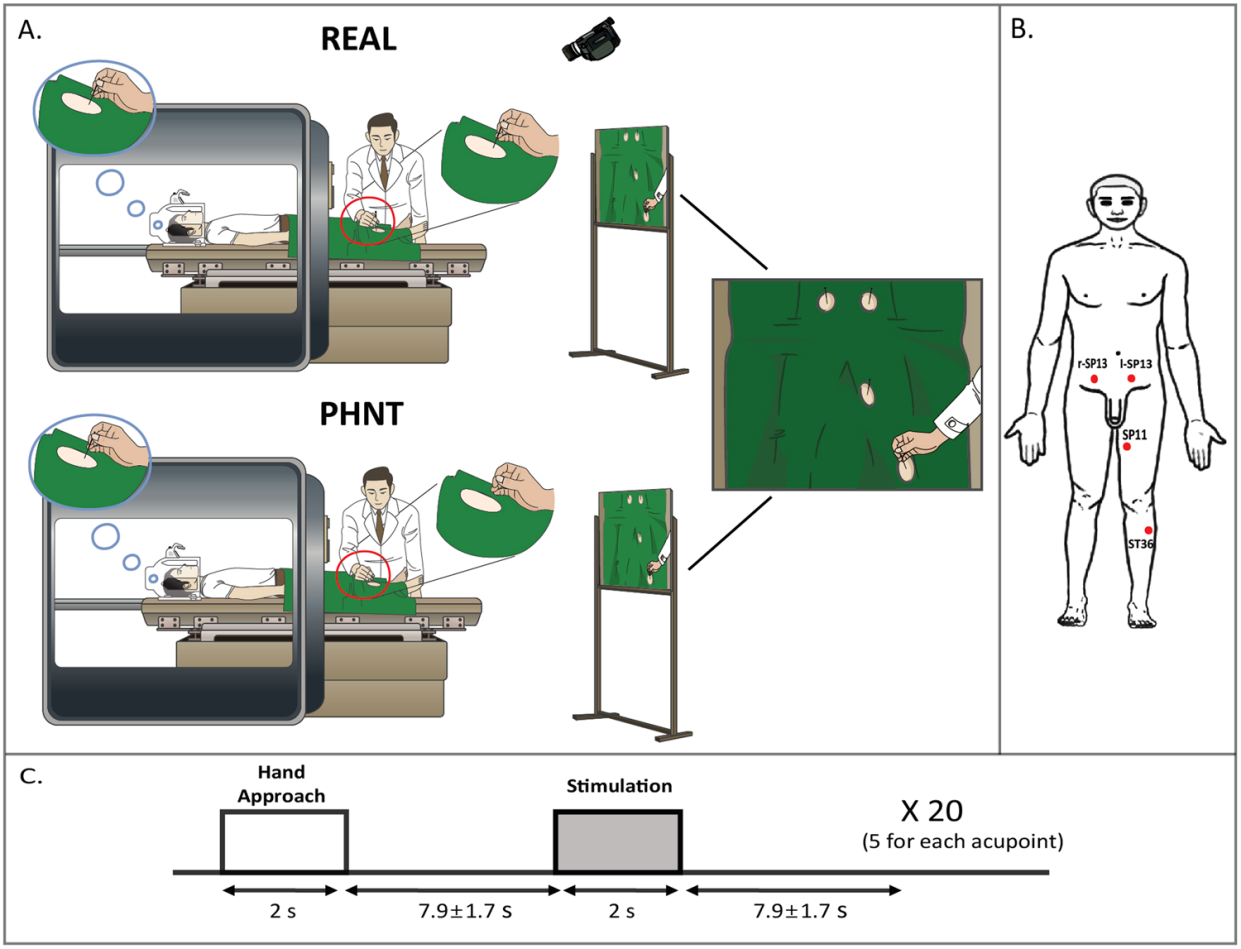
Experimental protocol. (**A**) Experimental setup for real (REAL) and phantom (PHNT) acupuncture stimulation. In REAL, participants got stimulated in four acupoints while watching the stimulation procedure via back projection, whereas in PHNT, no afferent stimulation was given to the participants. Instead, they watched a recorded video clip of a real stimulation. (**B**) The acupuncture stimulation point (acupoint) locations (ST36, SP11, and r, l-SP13). (**C**) The event-related experimental paradigm, which consisted of five acupuncture stimulations per acupoint; each stimulation was 2 sec long, and the average inter-stimulus interval was 7.9 ± 1.7 sec. n.b. REAL, real acupuncture; PHNT, phantom acupuncture.

Patients were randomized, i.e., parallel-group study, into real acupuncture (REAL, n = 33) and sham control phantom acupuncture (PHNT, n = 23) groups. The experiment consisted of four fMRI runs: 6-minute resting steady state run, 7-minute acupuncture run, and 6-minute pre- and post-acupuncture steady pain state runs. (In this paper, we focus on the acupuncture run. The rest of the data will be discussed in a separate paper). Figure 2A shows the experimental setup and paradigm for the REAL/PHNT run. In the acupuncture stimulation run, LBP patients experienced either real acupuncture (REAL) or phantom acupuncture (PHNT), which we devised in a previous study^6^.

Patients lay supine inside the MRI machine, and they were told that they would receive real acupuncture treatment during this experiment. As a preparatory procedure, a camera inside the scanning room was turned on, and the image from the camera was shown to patients through a beam projector while the experimenter/acupuncturist prepared the acupuncture stimulation. This procedure allowed the patients to build a link (main source of needling credibility) between the displayed video and their own body. Once the acupuncture stimulation run was ready, the camera was turned off until the REAL or PHNT run started for next experimental procedure, needle insertion. Visual access to their distal body was restricted to prevent them from seeing the type of intervention applied at the acupoints in their periphery during the acupuncture stimulation run.

For the REAL group, acupuncture stimulation with sterilized non-magnetic needles (0.3 mm × 30 mm, stainless steel, DongBang Co., Korea) was applied by a licensed acupuncturist who had more than 10 years of clinical experience and was trained to work in an fMRI experimental environment/protocol. Needles were inserted ~2–3 cm deep into four acupoints: bilateral SP13, left SP11, and left ST36 (Fig. 2B). These acupoints were chosen based on their known clinical effectiveness and relatively easy access to the acupuncturist during fMRI scanning. Once the needles were inserted, the acupuncturist manipulated them to induce a specific acupuncture sensation (*de-qi*) and ensure that the needles were placed correctly. The real acupuncture stimulation was applied manually by ±180° rotation of the needles at ~1 Hz during 2-second duration events in an event-related (ER) design (inter-stimulation interval = 7.9 ± 1.7 seconds) (Fig. 2C). Auditory cues were generated using in-house computer software (MATLAB, The MathWorks, MA, USA) and relayed to the acupuncturist via MR-compatible headphones to synchronize the acupuncture stimulation with the stimulation paradigm. During the run, the acupuncture stimulation procedure was video-recorded (with the same camera used in the preparatory procedure) and simultaneously displayed to the subject via a screen that reflected a projector outside the scanner. This was done to enhance participants’ belief in the acupuncture stimulation (i.e., needling credibility) (Fig. 2A).

For the PHNT run, the experiment deviated slightly from the REAL protocol to provide needling credibility without any somatosensory stimulation. Before the PHNT run started, the exact same preparatory procedure was performed, turning on the camera and building the video–body link. For PHNT, however, the acupuncturist neither inserted nor stimulated the needles. The acupuncturist only approached the acupoints with his hand and mimicked the stimulation without touching any body part of the patients, following the auditory cues for stimulation in the same sequence as in the REAL protocol. The video clip, which was recorded previously from the REAL group, was carefully chosen to match the body shape and skin appearance of each patient and displayed to the patient to create an illusion of needle insertion and stimulation (i.e., needling credibility), as shown in Fig. 2A. The REAL group experienced somatosensory needling stimulation with needling credibility enhanced by visual stimulation, and the PHNT group experienced only the needling credibility from the visual stimulation without any somatosensory needling stimulation.

During the acupuncture stimulation run (PHNT and REAL), a trigger pulse signal from the scanner kept the fMRI data synchronized with the physiological data acquisition (see physiological data acquisition section for more details) and the auditory cue (for the acupuncture stimulation run). This process ensured the synchronization of the discrete acupuncture stimulation with autonomic/physiological and imaging data acquisition.

### Retrospective Re-classification According to Needling Credibility Interview

After finishing the experiment, we retrospectively reclassified the PHNT group into two subgroups, PHNT credible patients (who had needling credibility) and PHNT non-credible patients (who did not) using an in-depth interview and a questionnaire that assessed the credibility of the acupuncture procedure— whether they believed that they experienced real acupuncture or recognized the procedure as a sham for any reason during the experiment. The questionnaire items included questions about the thickness of the inserted needle (e.g., 0.05 mm, 0.2 mm, 1 mm and 2 mm), the depth of insertion (e.g., 1 cm, 2 cm, 3 cm, and 4 cm), the rotation rate of the needle (e.g., 4 Hz, 2 Hz, 1 Hz, and 0.8 Hz) to make sure if the patients paid attention to the stimulation, and whether they thought (due to any reason) that the acupuncturist did not insert or rotate the needles. Patients who reported any doubt or realized that the procedure was not truly happening to themselves (e.g., needles were not inserted or stimulated, the body part shown on the screen was not their own) during the experiment were considered as PHNT non-credible. In the analysis of this paper, we include only the PHNT-credible patients, calling them the PHNT group for convenience.

### Behavioral Measures

Before the fMRI runs, we collected several questionnaires from the patients to investigate individual differences that might influence the results of the experiment. We used a perception of bodily sensation^35^ questionnaire to assess individuals’ body sensitivity to the external environment, which might be related to person’s ability to discriminate acupuncture stimulation (REAL vs. PHNT). In addition, we used the State-Trait Anxiety Inventory (STAI) for adults^36^ to measure anxiety levels, which could affect the brain and autonomic responses^37^. We collected acupuncture efficacy expectation scores^38^ to quantify subjects’ beliefs about acupuncture treatment effectiveness, which is known to be an important factor in the placebo effect. This latter questionnaire contains 36 items about three aspects of acupuncture effectiveness: general belief in acupuncture efficacy; scientific credibility, which addresses the scientific value of acupuncture treatment, for example, “I believe acupuncture treatment is a scientific treatment”; and adverse events that could be caused by the procedure, along with the physical experience of acupuncture. These scores use a five-point Likert-type scale (1: strongly disagree, 5: strongly agree). We calculated an average belief in acupuncture effectiveness score to represent the overall subjective belief in acupuncture as an effective treatment. Higher value of this score reflects a stronger belief in acupuncture effectiveness. After the acupuncture stimulation, we asked patients to rate the intensity of acupuncture sensations (aching, pressure, soreness, fullness, heaviness, warmth, numbness, coolness, tingling, and dull pain) on a scale from 0 (no perceived tactile sensation) to 10 (very intense tactile sensation) using an in-house Korean version of the Massachusetts General Hospital Acupuncture Sensation Scale (MASS) Index, calculated from the weighted sum of the sensation values^39^. The significance of different sensation intensities for each stimulation group were calculated using one sample *t*-test, significance at *p* < 0.05 (SPSS v. 10.0.7, Chicago, IL, USA). Pain intensity in the low back was measured using an 11-point VAS (0: no pain, 10: maximum imaginable pain) before and after the acupuncture stimulation run for both groups (REAL and PHNT). Change in pain intensity (ΔVAS) was calculated as a main outcome.

### Brain MRI Data Acquisition

MRI data were collected using a 3.0 Tesla Philips Achieva system (Philips Medical Systems, The Netherlands) with an 8-channel head coil. Restriction of head movements and subject comfort was provided by foam-padding the coil. Participants were instructed to lie in a supine position inside the scanner and were given earplugs to block the MR gradient switching noise. Anatomical brain imaging was collected using a three-dimensional *T*_1_-weighted pulse sequence (magnetization prepared rapid acquisition gradient-echo (MPRAGE), repetition time (*T*_*R*_)/echo time (*T*_*E*_) = 9886/4.59 *ms*, field of view (FOV) = 256 × 256 *mm*^2^, flip angle = 8°, voxel size = 1 × 1 × 1 *mm*^3^) for anatomical localization. FMRI data were collected using a two-dimensional ${T}_{2}^{\ast }$-weighted echo planar imaging (EPI) pulse sequence (*T*_*R*_/*T*_*E*_ = 2000/35 *ms*, FOV = 230 × 230 *mm*^2^, flip angle = 90°, voxel size = 2.875 × 2.875 × 4 *mm*^3^, matrix = 80 × 80, 34 interleaved axial slices, sensitivity-encoded (SENSE) factor = 2, and 200 whole brain volumes) for the acupuncture stimulation run. We started the fMRI run with four dummy volumes, which we discarded to allow for *T*_1_ equilibration effects.

### Physiological Data Acquisition

Throughout the fMRI scanning run, we acquired physiological data: skin conductance response (SCR), electrocardiogram (ECG), and respiratory signals, from different end organs using the Powerlab system (ML800, AD Instruments, Australia) at a 1 kHz sampling rate. The SCR signal was acquired from the subject’s left index and middle finger using MRI-compatible Ag/Ag-Cl electrodes (MLT117F, GSR Amp, AD Instruments, Australia). The ECG was collected using Ag/AgCl electrodes (Kendall, Covidien, USA) and filtered using a 60 Hz notch filter (ML132, BIO Amp, AD Instruments, Australia). The respiratory signals were acquired using an MRI-compatible in-house pneumo-belt system in accordance with the system devised by Binks *et al*.^40^.

### FMRI Data Preprocessing

FMRI brain data preprocessing and analysis were performed using validated, conventional software: AFNI^41^, FSL (FMRIB’s Software Library, Oxford, UK)^42^, FreeSurfer (Martinos Center for Biomedical Imaging, Boston, MA, USA)^43^, and SUMA (National Institute of Mental Health, Bethesda, MD, USA)^44^. Cardio-respiratory artifacts caused by cardiac pulsatility and respiration were mitigated in the fMRI data using the RETROICOR algorithm^45^. Head movements were corrected using a motion correction algorithm (FSL-MCFLIRT) with affine rigid-body transformation. Brain extraction for functional and anatomical data was performed using FSL-BET^46^ and mri_watershed (FreeSurfer), respectively^47,48^. Cortical surface reconstruction was done to ensure accurate anatomical-functional co-registration using the bbrigester tool (FreeSurfer)^49^. Functional data were spatially smoothed using a Gaussian kernel of 5 *mm* full-width at half-maximum, and high-pass temporal filtering (cut-off frequency, f = 0.05 Hz) was performed. Preprocessed functional and structural data were then registered to standard Montreal Neurological Institute (MNI) space using FNIRT (FMRIB’s nonlinear image co-registration tool). This was done into two steps. First, the initial affine normalization of the each subject’s structural image to the reference MNI-template was performed using FLIRT (FMRIB’s linear image co-registration tool). Then, this was fed to FNIRT to perform the overall transformation (i.e., computing the transformation matrix necessary to map the anatomical image to the reference image). Finally, this transformation matrix was used to normalize the native statistical outputs to the reference MNI space image for group analysis.

### Brain Response to Hand Approach and Acupuncture Stimulation Events

To get the brain responses to hand approach and acupuncture stimulation (REAL and PHNT) events, we constructed a general linear model (GLM, FSL-FEAT) with separate regressors convolved with a canonical double gamma hemodynamic response function for each event. Nuisance regressors from the cardiac and respiratory response functions were added to this GLM to eliminate any residual cardio-respiratory artifacts from the brain data^45,50,51^. In addition, we added motion-related nuisance regressors to the constructed GLM: the extracted time-course signal from ventricles and six head motion parameters (FSL-MCFLIRT).

### Group fMRI Data Analysis

To perform group analyses, we transformed individual GLM outputs (i.e., copes and varcopes) into MNI space (FNIRT). We calculated the group main effect of real and phantom acupuncture stimulation and hand approach events using one sample *t*-test in a mixed effects statistical model (FLAME1 + 2, FSL-FEAT). Common brain responses to REAL and PHNT and the differences between them were calculated using conjunction and difference analyses, respectively (FLAME1 + 2, FSL-FEAT). Difference maps between acupuncture stimulation (STIM) and hand approach (HAND) events were performed using paired *t*-test (FLAME1 + 2, FSL-FEAT) for both groups. All of the fMRI results were cluster-corrected for multiple comparisons at *Z* = 2.3, *p* < 0.05.

To investigate the correlation between brain responses to acupuncture stimulation (REAL and PHNT) and outcome measures (e.g., acupuncture efficacy expectation scores, MASS Index, ΔVAS), and ANS metrics (e.g., HR, SCR), we performed analysis of covariance (ANCOVA, FSL-FEAT, cluster-corrected for multiple comparisons, *Z* = 2.3, *p* < 0.05).

### Physiological Data Analysis

Peripheral ANS data (SCR and HR) were synchronized to each other and to the fMRI data using the MR-generated trigger pulse^8^. SCR, representing the sudomotor activity, was quantified as the area under curve (AUC, *μ*S/s) from a 6 sec window after stimulus onset^52^. Physiological signals were averaged relative to the onset time of each acupuncture stimulation event, and with respect to a baseline window (2-sec) preceding each event. HR metric was calculated as the maximum decrease relative to this baseline (within 6 sec window). SCR and HR data were individually compared between REAL and PHNT groups following acupuncture stimulation events using a Student’s *t*-test (SPSS, Chicago, IL). Cross-correlation analyses between ANS outflow metrics (SCR and HR) following acupuncture stimulation events were also performed using Pearson’s correlation coefficient.

## Results

After the retrospective reclassification of LBP patients in the PHNT group, four patients were excluded as non-credible (i.e., needling credibility was reported in $\simeq$82% of the PHNT group) (Fig. 1). Out of the REAL group, we excluded five LBP patients due to excessive head motion and incomplete data. Patients were thus divided into two final groups: 28 REAL patients (12 female, age = 38.7 ± 13.1 years old, mean ± STD) and 19 PHNT patients (10 female, age = 39.5 ± 13.7 years old).

We found no significant differences between the REAL and PHNT groups in baseline data: age (*p* = 0.83), perception of bodily sensation (REAL = 40.68 ± 7.58, PHNT = 41.37 ± 7.45, *p* = 0.99), STAI (STAI-state: REAL = 37.15 ± 7.98, PHNT = 33.82 ± 8.75, *p* = 0.15; STAI-trait: REAL = 37.69 ± 7.69, PHNT = 35.9 ± 9.24, *p* = 0.44), and belief in acupuncture effectiveness score (REAL = 3.67 ± 0.38, PHNT = 3.77 ± 0.37, *p* = 0.36).

### Psychophysics and Clinical Outcome

Consistent with our previous study^6^, the PHNT group reported significant acupuncture sensations (e.g., aching, heaviness) (Table 1). The REAL group also reported significant acupuncture sensations (e.g., aching, soreness, deep pressure, heaviness, fullness, tingling, dull pain, throbbing, and sharp pain) (Table 1). In addition, we found a significant VAS decrease when comparing before and after stimulation scores in the PHNT group, but not in the REAL group (Table 1).

**Table 1:** Psychophysics and clinical outcome comparison between both groups.

Outcome Measure	REAL	PHNT	REAL Vs. PHNT
**Acupuncture sensation intensity (0–10)**			
Aching	2.29 ± 1.87, *p* < 0.05	0.44 ± 0.81, *p* < 0.05	*p* < 0.01
Soreness	2.04 ± 1.70, *p* < 0.05	0.33 ± 0.97, *p* = 0.15	*p* < 0.01
Deep pressure	2.62 ± 1.88, *p* < 0.05	0.52 ± 1.20, *p* = 0.07	*p* < 0.01
Heaviness	2.44 ± 1.84, *p* < 0.05	0.57 ± 1.23, *p* < 0.05	*p* < 0.01
Fullness	1.86 ± 1.56, *p* < 0.05	0.25 ± 0.72, *p* = 0.14	*p* < 0.01
Tingling	2.21 ± 1.85, *p* < 0.05	0.26 ± 0.96, *p* = 0.24	*p* < 0.01
Dull pain	1.42 ± 1.29, *p* < 0.05	0.28 ± 0.72, *p* = 0.10	*p* < 0.01
Throbbing	0.94 ± 1.15, *p* < 0.05	0.20 ± 0.48, *p* = 0.08	*p* < 0.01
Sharp pain	2.06 ± 1.79, *p* < 0.05	0.26 ± 0.71, *p* = 0.12	*p* < 0.01
Spreading	1.46 ± 1.56, *p* < 0.05	0.46 ± 1.13, *p* = 0.08	*p* < 0.05
MASS index	2.88 ± 1.80, *p* < 0.05	0.61 ± 1.30, *p* < 0.05	*p* < 0.01
**Low back pain intensity (0–10)**			
Pre-VAS	2.79 ± 1.63, *p* < 0.05	3.70 ± 1.49, *p* < 0.05	*p* < 0.05
Post-VAS	2.64 ± 1.34, *p* < 0.05	3.31 ± 1.70, *p* < 0.05	*p* = 0.25
Δ VAS	−0.61 ± 1.66, *p* = 0.62	−0.56 ± 1.27, *p* < 0.05	*p* = 0.30

### ANS Response to REAL and PHNT Acupuncture: HR and SCR

**Figure 3:**
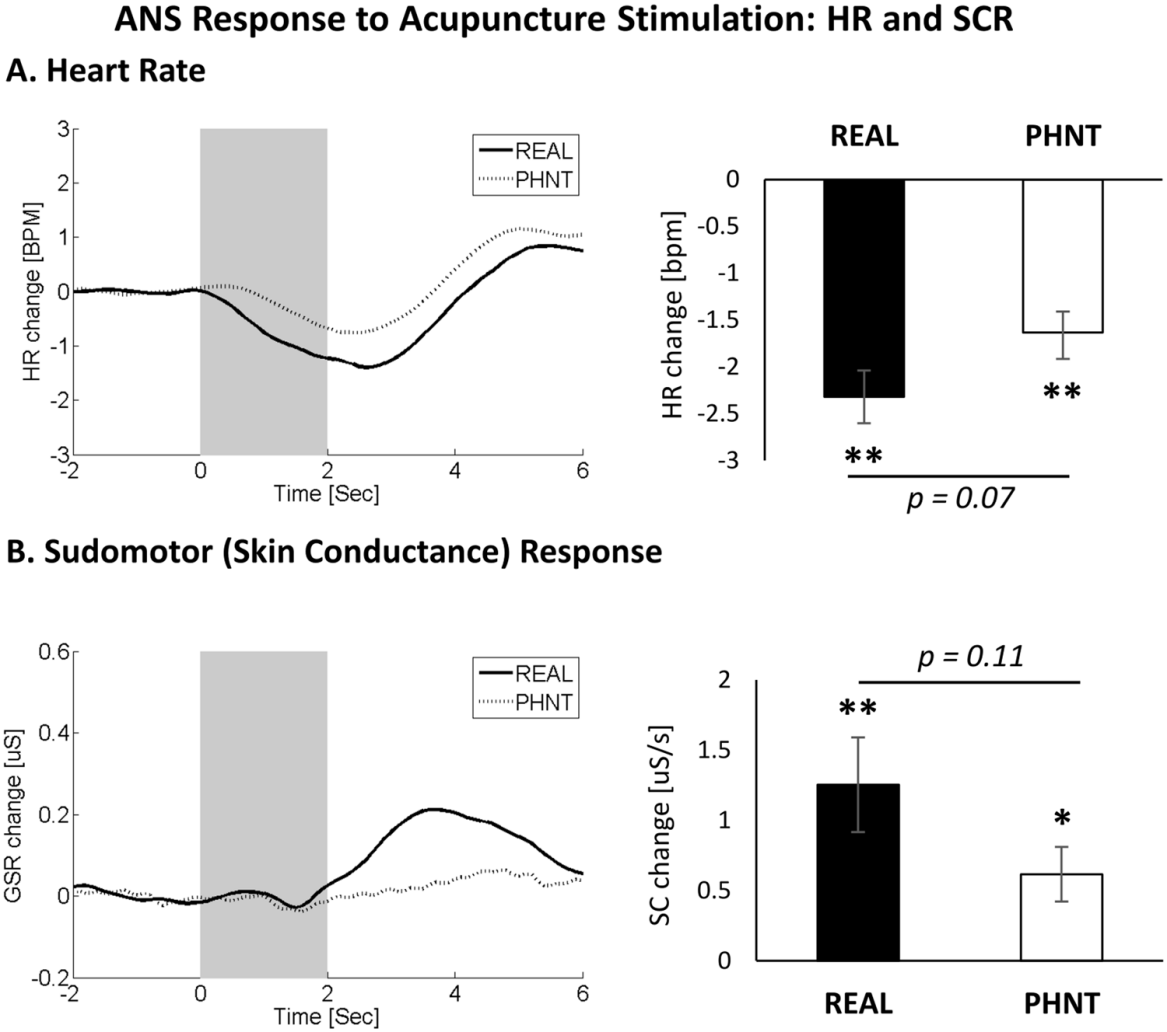
ANS response to acupuncture stimulation. (**A**) Decreased heart rate was noted following both of REAL and PHNT acupuncture simulation events. (**B**) Increased phasic sudomotor (skin conductance) response was also noted in response to REAL and PHNT acupuncture stimulation events. N.b. * < 0.01, ** < 0.001. Error bars represent standard error of the mean.

HR and SCR responses to acupuncture stimulation were calculated for both REAL and PHNT groups (Fig. 3). Averaged data showed a HR decrease and SCR increase following acupuncture stimulation events in both groups (i.e., REAL and PHNT). Maximum HR decrease showed no significant difference between both groups (REAL vs. PHNT, *p* = 0.07, where REAL = −2.3 ± 0.28 BPM, *p* < 0.01, PHNT = −1.6 ± 0.22 BPM, *p* < 0.01). The phasic SCR increase also showed no significant difference between both groups (REAL vs. PHNT, *p* = 0.11, where REAL = 1.25 ± 0.33 *μ*S/s, *p* < 0.001, PHNT = 0.62 ± 0.19 *μ*S/s, *p* < 0.01).

Analysis of cross-correlation between HR and SCR showed no significant correlation between SCR and HR for both groups (SCR/HR: REAL, *r* = 0.09, *p* > 0.5; PHNT, *r* = −0.14, *p* > 0.5), i.e., events eliciting stronger SCR increase were not more likely to elicit strong HR decrease. In addition, cross-correlation analysis between ANS data and other outcomes revealed no significant correlation (HR/MASS index, SCR/MASS index, MASS index/ΔVAS, HR/ΔVAS, SCR/ΔVAS, Belief/ΔVAS, and Belief/MASS index) in both groups (*r* < 0.3, *p* > 0.1). Interestingly, in PHNT but not REAL, we found significant correlation between belief in acupuncture effectiveness and the induced skin conductance activity (SCR/Belief: REAL, *r* = −0.17, *p* = 0.42; PHNT, *r* = 0.47, *p* = 0.05).

### Brain Responses to Hand Approach Events

**Figure 4:**
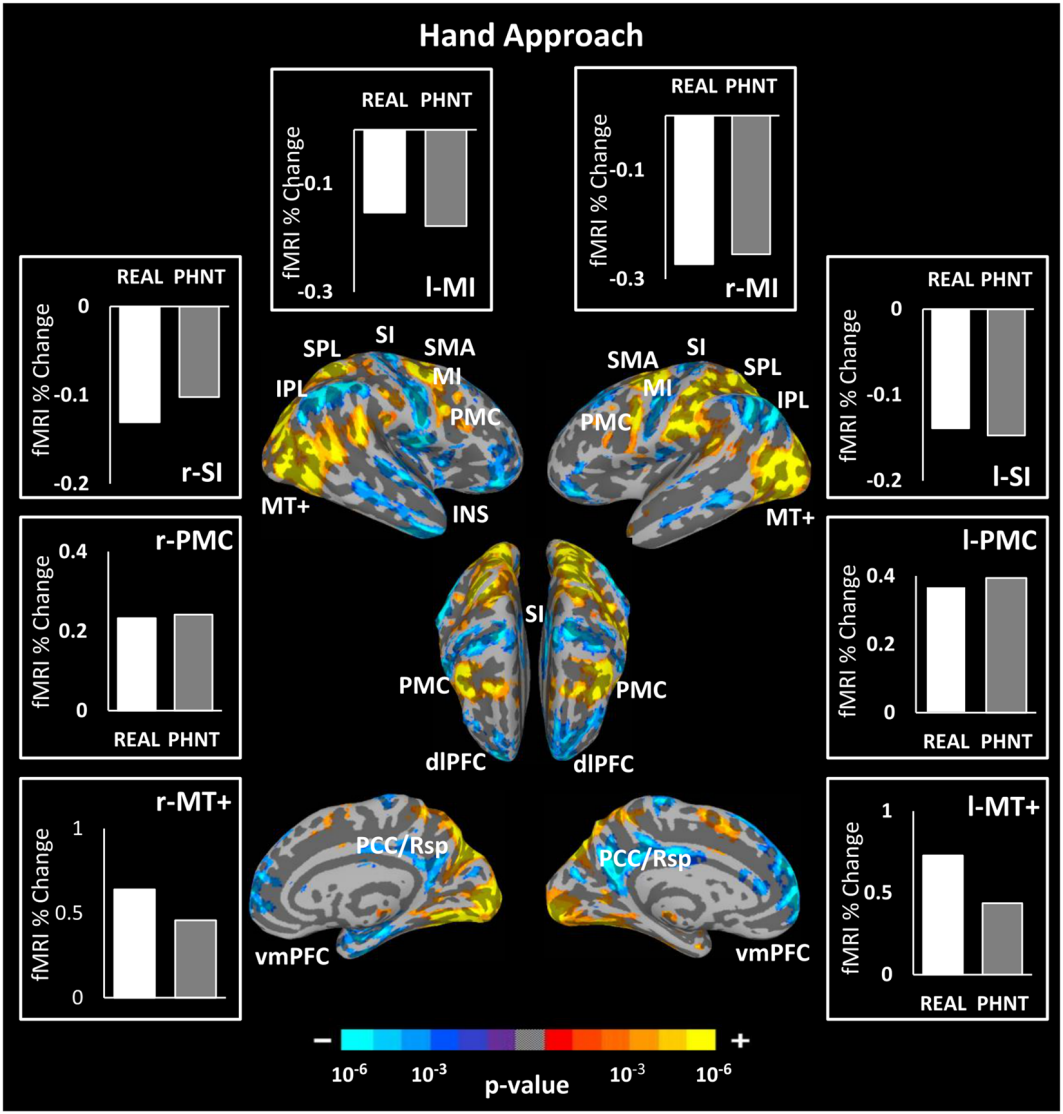
Brain response group map of hand approach events. Both groups revealed similar brain responses to hand approach events including significant signal increase in the PMC, SMA, MT+, SPL and significant signal decreases in the MI, SI, vmPFC, PCC and IPL. Bar figures represent the mean of the group activations extracted from an ROI (4 mm diameter sphere) around the activation peak in the corresponding brain area. n.b. IPL, inferior parietal lobule; SI, primary somatosensory cortex; MT+, middle temporal complex; PCC/Rsp, posterior cingulate cortex/retrosplenial cortex; dlPFC, dorsolateral prefrontal cortex; ACC, anterior cingulate cortex; vmPFC, ventromedial prefrontal cortex; SPL, superior parietal lobule; PMC, premotor cortex; SMA, supplementary motor area; INS, insular cortex.

Since hand approach events carries visual information of approaching the acupuncturist’s hand toward the needles, its brain responses is expected to be identical in both groups. This was confirmed by the difference map between hand approach events for REAL vs. PHNT groups which revealed no significant difference. Group map of hand approach events after combining both groups demonstrated significant BOLD signal increase in the MT+, PMC, SMA and SPL, and signal decrease in the MI, SI, vmPFC, IPL, and PCC (Fig. 4).

### Brain Responses to REAL and PHNT Stimulation

**Figure 5:**
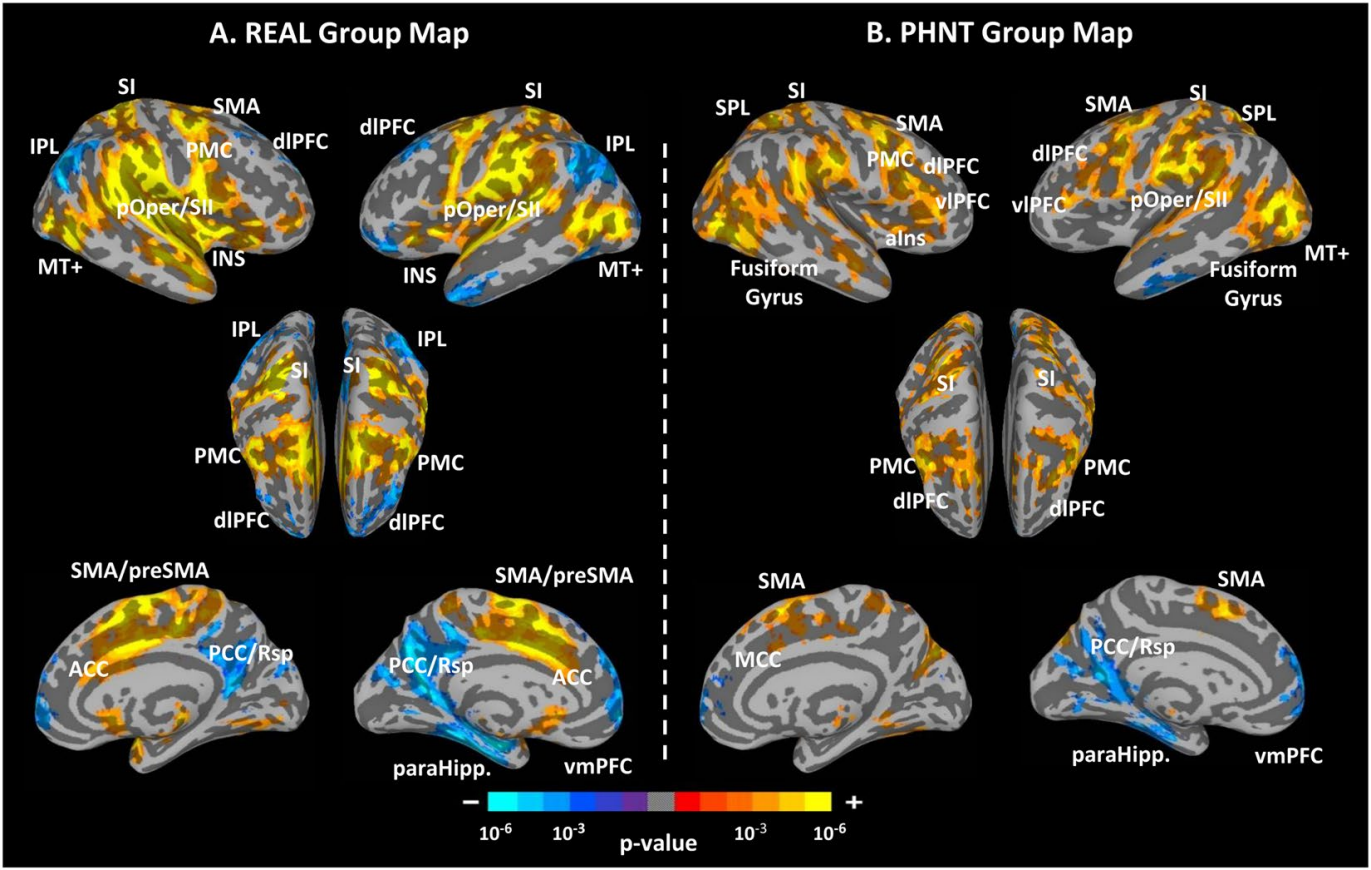
Brain response group maps for real (REAL) and phantom (PHNT) acupuncture stimulation. (**A**) REAL elicited significant activation in the pIns, ACC, SI, pOper/SII, MI, SMA, STG, paraHipp., vlPFC (BA 47), PMC and MT+, and significant deactivation in the IPL and dlPFC (BA 8, 10). B. PHNT elicited significant activation in the vlPFC (BA 44, 45), dlPFC (BA 46), SPL, SI, pOper/SII, PMC, MT+, STG, fusiform gyrus and MCC. n.b. pOper/SII, parietal operculum/secondary somatosensory cortex; vlPFC, ventrolateral prefrontal cortex; ACC, anterior cingulate cortex; INS, insular cortex; aIns, anterior insular cortex.

Group mean analyses were performed for both groups separately to investigate the brain response to each condition: REAL and PHNT. Our results demonstrate significant blood-oxygen-level dependent (BOLD) fMRI signal increases in response to real acupuncture (REAL) stimulation in (1) somatosensory/motor processing regions: bilateral posterior insula (pIns), primary somatosensory cortex (SI), parietal operculum/secondary somatosensory cortex (pOper/SII), anterior cingulate cortex (ACC), middle cingulate gyrus (MCC), supplementary motor area (SMA), premotor cortex, thalamus, putamen, and caudate nucleus, (2) placebo/pain analgesic/reward regions: vlPFC (BA 47), periaqueductal grey (PAG), and nucleus accumbens (NAC), (3) visual processing/association regions: middle temporal complex (MT+), temporoparietal junction (TPJ), middle occipital gyrus (MOG), superior temporal gyrus (STG), and supramarginal gyrus (SMG). We found significant BOLD signal decreases in the (1) dlPFC (BA 8, 10) and (2) default mode network (DMN): posterior cingulate cortex/retrosplenial cortex (PCC/Rsp), precuneus, medial prefrontal cortex (mPFC), parahippocampus (paraHipp), inferior parietal lobule (IPL), and angular gyrus (AG) (Fig. 5A, Table 2).

**Table 2:** Main effect map summary for real acupuncture (REAL) group.

REAL Group Mean	Size (# Voxels)	Side	Anatomical Location	x (mm)	y (mm)	z (mm)	Peak (z-score)
Activation	43523	L	Superior Temporal Gyrus	−60	−28	16	6.91
		R	Parietal Operculum/SII	58	−18	16	6.81
		R	Postcentral Gyrus (SI)	24	−40	66	6.62
		L	Superior Temporal Gyrus	−60	6	−2	6.58
		R	Middle Temporal Gyrus	50	−64	6	6.41
		L	Middle Occipital Gyrus	−56	−70	4	6.33
		L	Postcentral Gyrus (SI)	−22	−40	70	6.26
		R	Superior Temporal Gyrus	58	2	4	6.01
		R	Medial Frontal Gyrus (SMA)	0	2	48	6.00
		R	Superior Frontal gyrus (SMA)	0	−4	64	5.96
		L	Precentral Gyrus (MI)	−48	0	54	5.77
		L	Cingulate Gyrus (ACC)	−4	3	42	5.75
		R	Cingulate Gyrus (ACC)	4	12	40	5.63
		R	Middle Frontal Gyrus (PMC)	42	0	56	5.53
		L	Insula (a,m,pIns)	−41	−18	8	5.48
		R	Insula (a,m,pIns)	46	0	2	5.47
		R	Inferior Temporal Gyrus	46	−6	−20	5.36
		L	Cingulate Gyrus (MCC)	−16	−24	36	4.97
		L	Ventrolateral Prefrontal Cortex (BA 47)	−32	26	−2	4.96
		L	Postcentral Gyrus (SI)	−40	−42	62	4.88
		R	Cingulate Gyrus (MCC)	14	−24	42	4.62
		R	Thalamus	8	−24	−2	4.27
		R	Ventrolateral Prefrontal Cortex (BA 47)	42	46	2	4.08
		R	Lingual Gyrus	4	−74	−6	3.54
	1389	R	Cerebellar Tonsil	32	−54	−30	4.63
	422	R	Inferior Semi-Lunar Lobule	16	−68	−58	4.69
Deactivation	14626	L	Parahippocampal Gyrus	−26	−14	−24	−5.33
		L	Posterior Cingulate Gyrus(PCC/RSC)	−8	−60	14	−4.91
		R	Superior Temporal Gyrus	60	−60	26	−4.68
		L	Superior Parietal Lobule	−40	−72	50	4.64
		L	Fusiform Gyrus	−30	−39	−21	−4.48
		R	Precuneus	26	−56	34	−3.98
		L	Middle Temporal Gyrus	−49	2	−41	−3.98
		L	Inferior Parietal Lobule	−45	−69	49	−3.84
		R	Inferior Parietal Lobule	55	−51	47	−3.74
		R	Posterior Cingulate Gyrus(PCC/RSC)	5	−53	35	−3.49
		L	Thalamus	−24	−28	8	−3.48
	4119	L	Middle Frontal Gyrus (dlPFC, BA 8)	−26	28	58	−4.60
		L	Medial Frontal Gyrus (vmPFC)	−4	60	0	−4.36
		L	Medial Frontal Gyrus (dmPFC)	−2	58	24	−4.22
		L	Superior Frontal Gyrus (dlPFC, BA 10)	−22	64	14	−3.75
		R	Medial Frontal Gyrus (vmPFC)	5	65	6	−4.06
		R	Medial Frontal Gyrus (dmPFC)	5	51	30	−2.07
	931	R	Superior Frontal Gyrus (dlPFC, BA 10)	20	60	32	−4.50
		R	Middle Frontal Gyrus (dlPFC, BA 8)	33	40	42	−3.52
	804	L	Middle Frontal Gyrus (vlPFC)	−42	40	−14	−4.04
		L	Middle Frontal Gyrus (vlPFC)	−36	50	−2	−3.54
	118	L	Fusiform Gyrus	−42	−66	−18	−3.34
	80	L	Superior Frontal Gyrus	−2	28	64	−3.57
	25	L	Superior Frontal Gyrus (vlPFC)	−30	60	−14	−2.84
	22	R	Superior Parietal Lobule	10	−72	60	−2.99

The phantom acupuncture stimulation (PHNT) group also showed fMRI signal increases in (1) somatosensory/motor processing regions: right anterior insula (aIns), bilateral SI, pOper/SII, superior parietal lobule (SPL), MI, premotor cortex, and SMA, (2) placebo/pain analgesic regions: dlPFC (BA 46), vlPFC (BA 44 and 45), and PAG, and (3) visual processing/association regions: MT+, TPJ, STG, and fusiform area. We also found signal decreases in DMN regions of the PHNT group: left paraHipp, ventromedial prefrontal cortex (vmPFC), left PCC/Rsp, and precuneus (Fig. 5B, Table 3).

**Table 3:** Main effect map summary for phantom acupuncture (PHNT) group.

PHNT Group Mean	Size (# Voxels)	Side	Anatomical Location	x (mm)	y (mm)	z (mm)	Peak (z-score)
Activation	36637	L	Middle Occipital Gyrus	−52	−72	4	5.57
		L	Superior Parietal Lobule	−32	−54	56	5.39
		L	Parietal Operculum/SII	−60	−22	18	5.24
		R	Inferior Temporal Gyrus	48	−72	2	5.11
		R	Ventrolateral Prefrontal Cortex (BA 44)	44	6	30	5.10
		L	Precentral Gyrus (MI)	−46	−2	56	5.09
		R	Parietal Operculum/SII	60	−18	24	4.96
		R	Superior Frontal Gyrus (SMA)	4	12	56	4.83
		L	Postcentral Gyrus (SI)	−62	−22	42	4.77
		L	Middle Frontal Gyrus (PMC)	−38	0	46	4.77
		R	Postcentral Gyrus (SI)	26	−38	68	4.64
		R	Inferior Parietal Lobule	54	−32	26	4.62
		L	Precuneus	−14	−82	40	4.43
		R	Superior Occipital Gyrus	28	−78	26	4.37
		L	Postcentral Gyrus (SI)	−50	−28	52	4.32
		L	Ventrolateral Prefrontal Cortex (BA 44)	−43	4	33	4.28
		L	Inferior Semi-Lunar Gyrus	−8	−78	−44	4.23
		L	Superior Temporal Gyrus	−66	−42	18	4.17
		R	Superior Frontal Gyrus (SMA)	14	6	64	4.14
		L	Postcentral Gyrus (SI)	−28	−48	68	4.11
		L	Precentral Gyrus (MI)	−62	4	6	4.07
		R	Ventrolateral Prefrontal Cortex (BA 45)	58	30	6	3.97
		L	Cuneus	−28	−90	30	3.90
		R	Superior Frontal Gyrus (SMA)	10	−4	76	3.87
		R	Middle Temporal Gyrus	54	−10	−18	3.86
		R	Insula (mIns)	42	0	4	3.82
		L	Ventrolateral Prefrontal Cortex (BA 45)	−51	35	14	3.80
		R	Paracentral Lobule	10	−42	56	3.67
		L	Insula (mIns)	−46	−10	6	3.59
		R	Cingulate Gyrus (MCC)	10	4	44	3.48
		R	Inferior Occipital Gyrus	34	−84	−6	3.45
		L	Superior Temporal Gyrus	−56	10	−10	3.41
		L	Fusiform Gyrus	−42	−66	−18	3.08
		L	Middle Frontal Gyrus (dlPFC, BA 46)	−45	36	17	3.00
		R	Middle Frontal Gyrus (dlPFC, BA 46)	45	32	19	2.85
		R	Fusiform Gyrus	45	−56	−18	2.80
	1232	R	Uvula	8	−80	−46	4.67
	270	R	Lentiform Nucleus	18	−4	16	3.35
	130	R	Thalamus	6	−14	6	3.27
Deactivation	1804	L	Medial Frontal Gyrus(dmPFC)	−4	68	8	−3.85
		L	Medial Frontal Gyrus (vmPFC)	−4	46	−7	−3.42
		R	Medial Frontal Gyrus (dmPFC)	1	43	21	−3.30
		R	Medial Frontal Gyrus (vmPFC)	2	59	2	−3.20
	1629	L	Fusiform Gyrus	−30	−34	−16	−3.77
		L	Parahippocampal Gyrus	−34	−22	−28	−3.59
	1398	L	Posterior Cingulate (PCC/RSC)	−10	−56	10	−4.04
		L	Precuneus	−9	−69	22	−3.16
	382	L	Inferior Temporal Gyrus	−54	−12	−28	−3.55
		L	Middle Temporal Gyrus	−54	−19	−16	−2.96
	20	L	Precuneus	−20	−58	30	−2.84

### Common Brain Responses to REAL and PHNT Acupuncture

**Figure 6:**
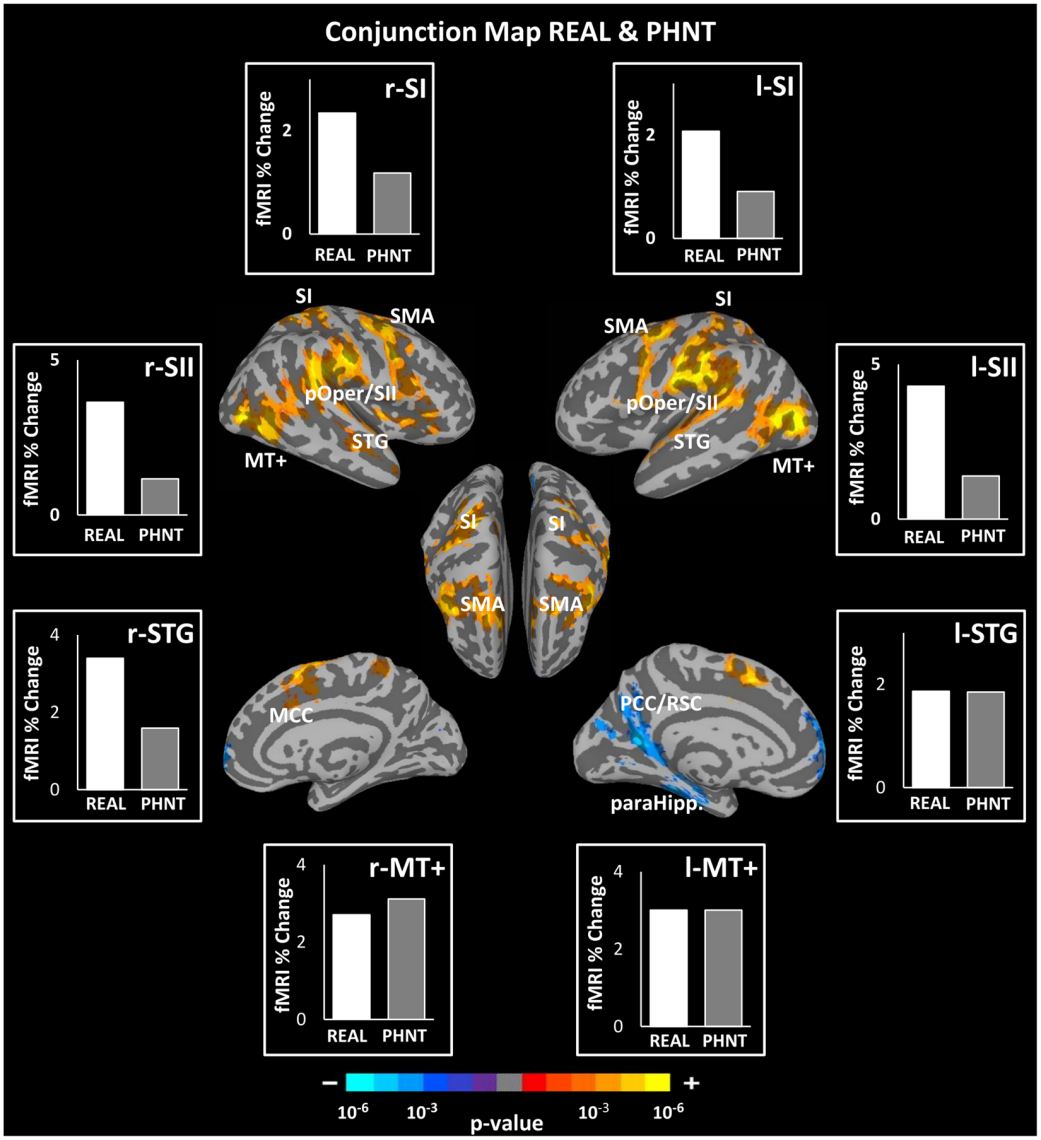
Conjunction map between real (REAL) and phantom (PHNT) acupuncture stimulation. Both conditions revealed significant activation in several brain regions: bilateral MI, SI, pOper/SII, PMC, STG, MT+, TPJ, and MCC, as well as the right mIns and aIns. Deactivation in the left vmPFC, PCC/Rsp, precuneus, and paraHipp was also found. Bar figures represent the mean of the group activation extracted from an ROI (4 mm diameter sphere) around the activation peak in the corresponding brain area.

Significant cortical activation in somatosensory/motor regions (right aIns, bilateral SI, pOper/SII, MI, premotor cortex, MCC) and visual processing/association regions (MT+, TPJ, and STG) was found as a common brain response in both the REAL and PHNT groups. We also found signal decreases in DMN regions (left vmPFC, PCC/Rsp, precuneus, and paraHipp) in both groups (Fig. 6, Table 4).

**Table 4:** Summary of conjunction map analysis between REAL and PHNT (REAL & PHNT).

Conjunction REAL & PHNT	Size (# Voxels)	Side	Anatomical Location	x (mm)	y (mm)	z (mm)	Peak (z-score)
Activation	20888	L	Middle Occipital Gyrus	−52	−72	4	5.57
		L	Parietal Operculum/SII	−60	−22	18	5.24
		L	Precentral Gyrus (MI)	−46	−2	56	5.09
		R	Parietal Operculum/SII	62	−18	26	4.91
		R	Inferior Temporal Gyrus	48	−70	2	4.87
		L	Postcentral Gyrus (SI)	−62	−22	42	4.77
		R	Inferior Frontal Gyrus (PMC)	48	6	32	4.65
		R	Postcentral Gyrus (SI)	26	−38	68	4.64
		R	Inferior Parietal Lobule	54	−32	26	4.62
		R	Middle Frontal Gyrus	30	−4	52	4.36
		L	Superior Parietal Lobule	−32	−50	60	4.24
		R	Insula (mIns)	42	0	4	3.82
		R	Insula (aIns)	38	24	4	3.69
		L	Precentral Gyrus (MI)	−50	−6	6	3.53
		L	Inferior Frontal Gyrus (PMC)	−56	12	4	3.49
		R	Cingulate Gyrus (MCC)	10	4	44	3.48
		L	Superior Temporal Gyrus	−64	−8	2	3.40
		R	Middle Temporal Gyrus	48	−16	−12	3.19
Deactivation	1073	L	Posterior Cingulate Gyrus /RSP	−10	−56	10	−4.04
	961	L	Medial Frontal Gyrus	−4	68	8	−3.85
	766	L	Parahippocampal Gyrus	−30	−34	−16	−3.77

### Differential Brain Responses to REAL and PHNT Acupuncture

**Figure 7:**
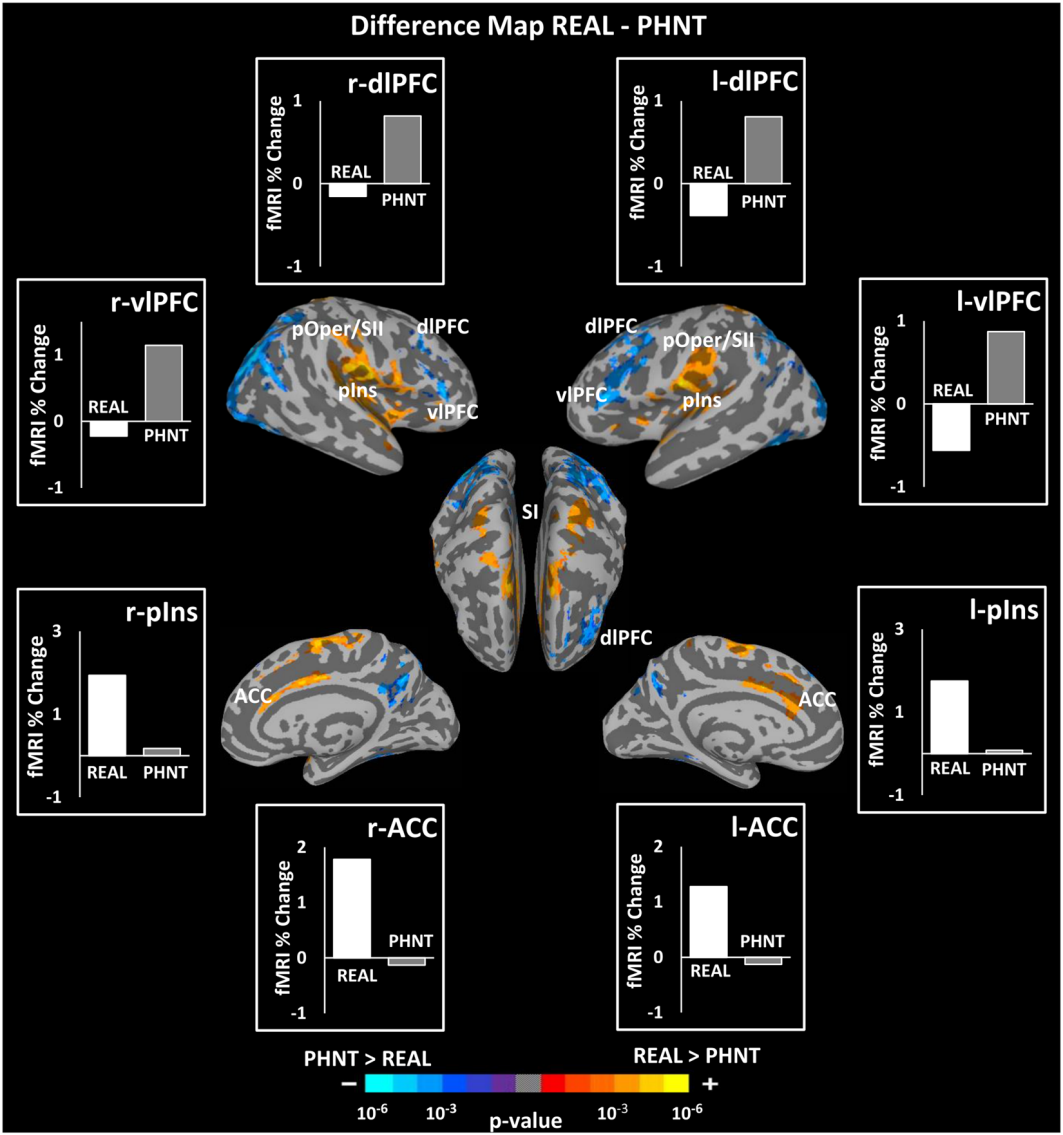
Difference map between real (REAL) and phantom (PHNT) acupuncture stimulation (REAL–PHNT). REAL elicited greater activation in the bilateral pIns, ACC, SI, pOper/SII, SMA, and deactivation in the IPL and dlPFC (BA 8) compared to PHNT, whereas PHNT elicited greater activation in the bilateral vlPFC (BA 44, 45) and dlPFC (BA 46) compared to REAL. Bar figures represent the mean of the group activation extracted from an ROI (4 mm diameter sphere) around the activation peak in the corresponding brain area.

Contrast between real and phantom acupuncture stimulation (i.e., REAL vs. PHNT) showed greater activation in the bilateral pIns, MI, SI, pOper/SII, aIns, ACC, MCC, STG and greater deactivation in the bilateral dlPFC (BA 8) and precuneus in the REAL group. On the other hand, the PHNT group showed greater activation in the bilateral vlPFC (BA 44, 45), dlPFC (BA 46), fusiform gyrus, and SPL (Fig. 7, Table 5).

**Table 5:** Summary of difference map analysis between REAL and PHNT (REAL - PHNT).

Difference REAL - PHNT	Size (# Voxels)	Side	Anatomical Location	x (mm)	y (mm)	z (mm)	Peak (z-score)
Activation	3722	R	Transverse Temporal Gyrus	50	−24	12	4.53
		R	Insula (mIns)	40	−18	−2	4.26
		R	Insula (pIns)	40	−20	14	3.81
		R	Postcentral Gyrus (SI, BA1,2)	60	−16	46	3.76
		R	Precentral Gyrus (MI)	60	6	8	3.63
		R	Postcentral Gyrus (SI)	22	−36	72	3.36
	2168	L	Medial Frontal Gyrus (SMA)	−2	−8	64	4.13
		R	Cingulate Gyrus(MCC)	2	−4	44	4.05
		R	Cingulate Gyrus (ACC)	4	12	40	3.64
		L	Cingulate Gyrus (MCC)	−8	8	36	3.48
		L	Cingulate Gyrus(ACC)	−4	26	25	3.02
	1376	L	Parietal Operculum/SII	−50	−16	12	4.25
		L	Insula (pIns)	−40	−18	10	4.01
		L	Transverse Temporal Gyrus	−54	−21	11	3.73
		L	Postcentral Gyrus (SI)	−54	−21	23	3.23
	647	L	Superior Temporal Gyrus	−60	8	−2	3.82
		L	Insula (a,mIns)	−38	17	−3	3.31
	537	L	Precentral Gyrus (MI)	−20	−26	76	3.89
		L	Postcentral Gyrus (SI)	−20	−38	70	3.67
		L	Superior Parietal Lobule	−22	−48	62	3.53
	298	R	Precentral Gyrus (MI)	20	−26	76	3.63
Deactivation	4414	R	Superior Occipital Gyrus	38	−78	28	−4.39
		R	Middle Occipital Gyrus	46	−64	−8	−4.16
		R	Ventrolateral Prefrontal Cortex (BA 44, 45)	58	30	6	−3.80
		R	Inferior Occipital Gyrus	38	−86	−14	−3.48
		R	Superior Temporal Gyrus	56	−56	18	−3.36
		R	Precuneus	24	−60	40	−3.33
		R	Superior Parietal Lobule	32	−50	48	−3.32
	2492	L	Middle Frontal Gyrus (dlPFC)	−34	16	22	−3.60
		L	Ventrolateral Prefrontal Cortex (BA 45)	−54	30	16	−3.53
		L	Middle Frontal Gyrus (dlPFC)	−52	28	28	−3.45
		L	Ventrolateral Prefrontal Cortex (BA 44)	−44	2	36	−3.34
	2416	L	Superior Parietal Lobule	−36	−58	52	−4.25
		L	Middle Occipital Gyrus	−36	−86	10	−3.63
	963	R	Inferior Semi-Lunar Lobule	22	−80	−44	−4.03
	868	L	Fusiform Gyrus	−42	−66	−18	−4.04
	735	R	Precuneus, PCC/RS	6	−54	38	−3.80
	522	L	Tuber	−36	−84	−40	−3.53
	314	R	Middle Frontal Gyrus (dlPFC)	44	16	34	−3.27
	219	R	Fusiform Gyrus	30	−38	−20	−3.09
	181	R	Ventrolateral Prefrontal Cortex	58	30	6	−3.80
	128	L	Superior Temporal Gyrus	−56	−62	26	−3.29
	95	L	Medial Frontal Gyrus (dmPFC)	−6	44	48	−3.18
	38	L	Superior Temporal Gyrus	−46	−52	18	−3.02

### Differential Brain Responses to REAL/PHNT Stimulation and Hand Approach Events

**Figure 8:**
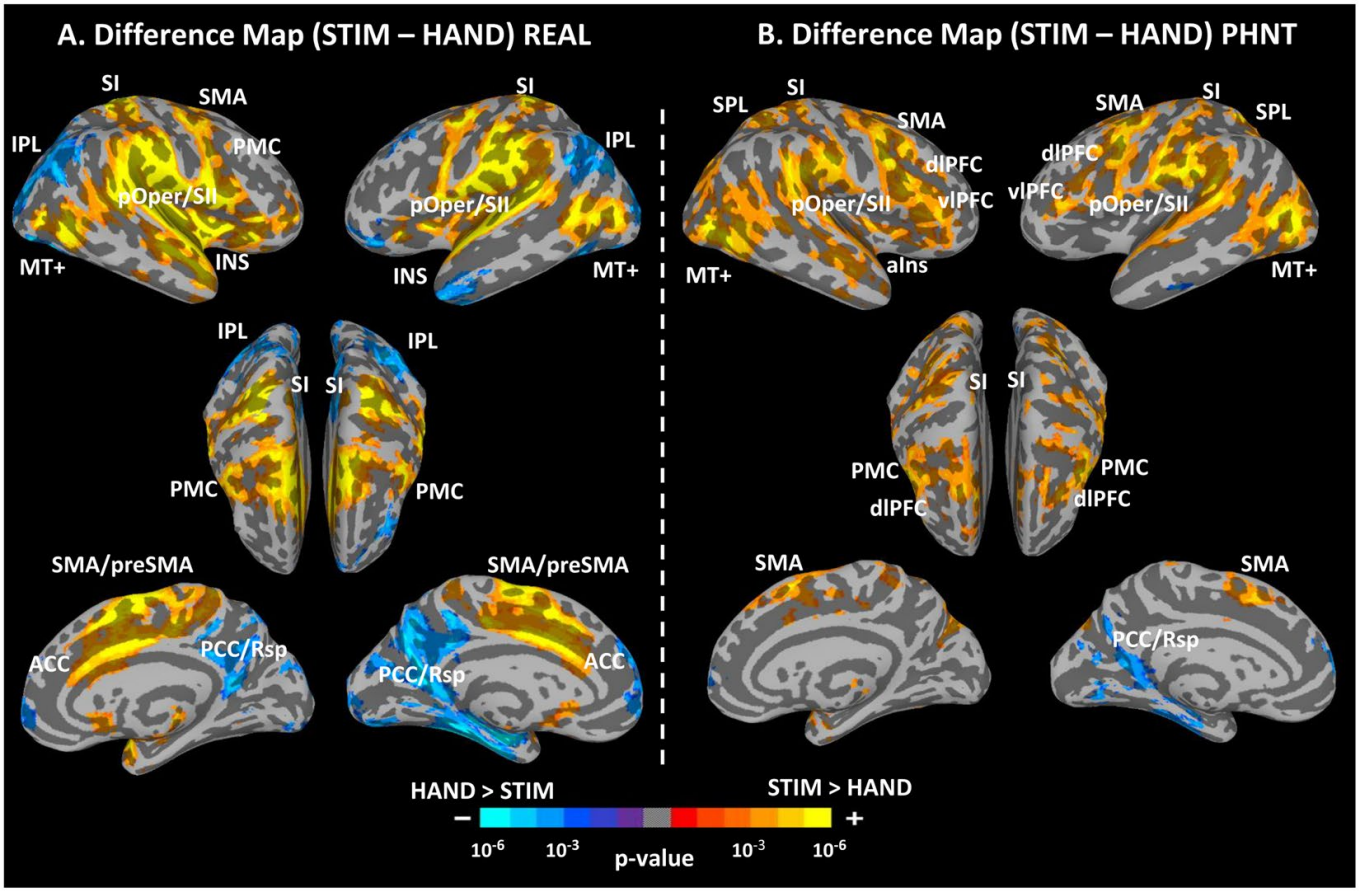
Difference maps between REAL/PHNT stimulation (STIM) events and hand approach (HAND) events for REAL and PHNT groups. (**A**) In REAL, STIM showed grater activation in the SI, pOper/SII, pIns, ACC, MI, SMA/preSMA, and MT+, and deactivation in the IPL and PCC/Rsp than HAND. (**B**) In PHNT, STIM showed greater activation in the SI, pOper/SII, vlPFC, dlPFC, SPL, MI, PMC, and MT+ than HAND.

Difference maps between STIM and HAND events were performed to investigate the brain responses unique to each kind of intervention in contrast to hand approach. In REAL, STIM showed greater activation in the bilateral SI, pOper/SII, pIns, ACC, MI, SMA/preSMA, and MT+, and deactivation in the bilateral IPL, vmPFC and PCC/Rsp than HAND (Fig. 8A). Interestingly, in PHNT, STIM showed greater activation in the bilateral SI, pOper/SII, vlPFC, dlPFC, SPL, MI, PMC, and MT+, and deactivation in the left PCC/Rsp than HAND (Fig. 8B).

### Brain Correlates with ANS Response, Belief in Acupuncture Effectiveness, ΔVAS, and MASS Index

**Figure 9:**
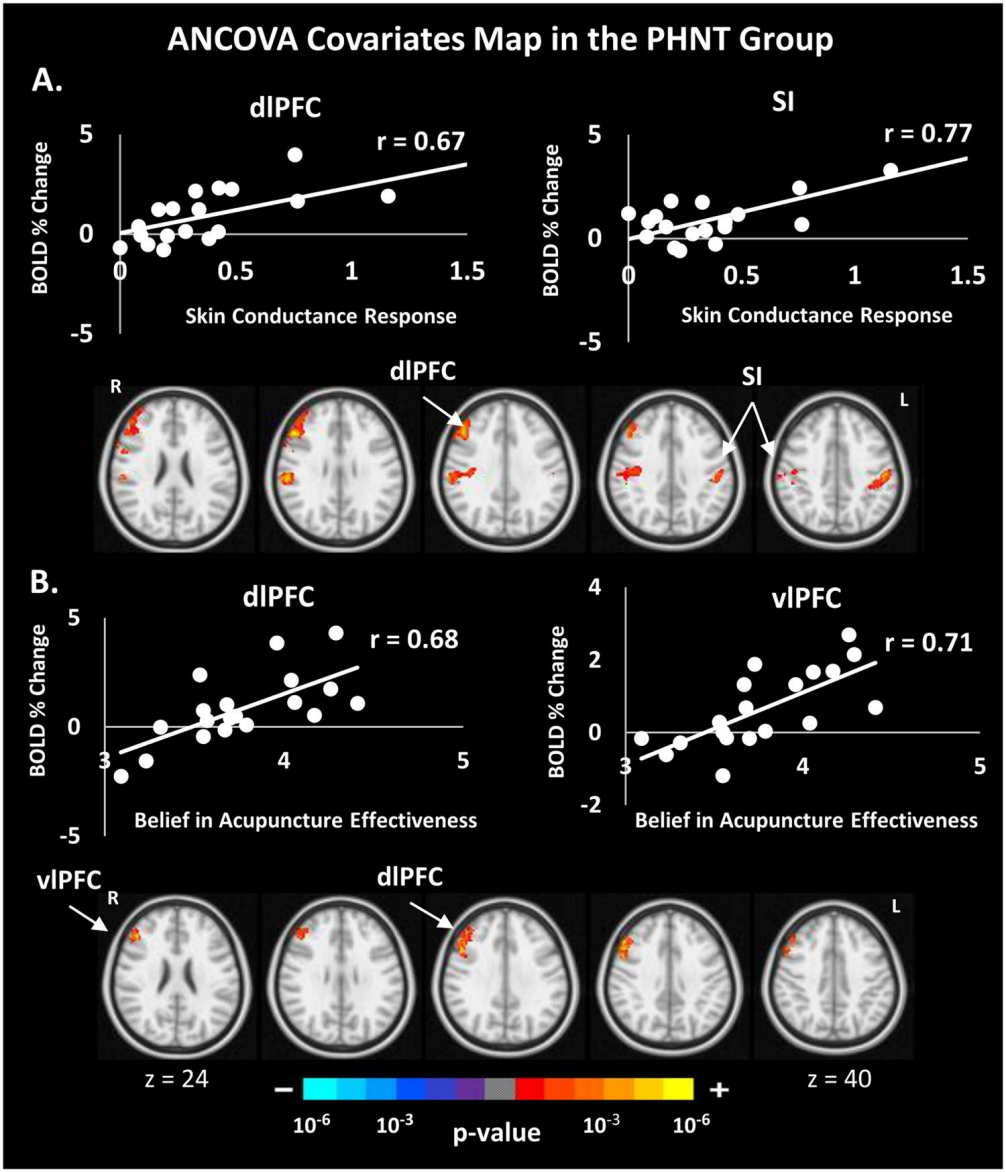
Correlation between subjective brain activity and the skin conductance response metric (**A**), and a score assessing belief in acupuncture effectiveness (**B**) for the PHNT group. The PHNT group, but not the REAL, has a significant correlation between sudomotor activity and fMRI signal in the right DLPFC and SI (**A**), and a significant correlation between subjective fMRI signals in the right DLPFC and VLPFC and average score assessing the expectation and belief in acupuncture effectiveness (**B**).

**Figure 10:**
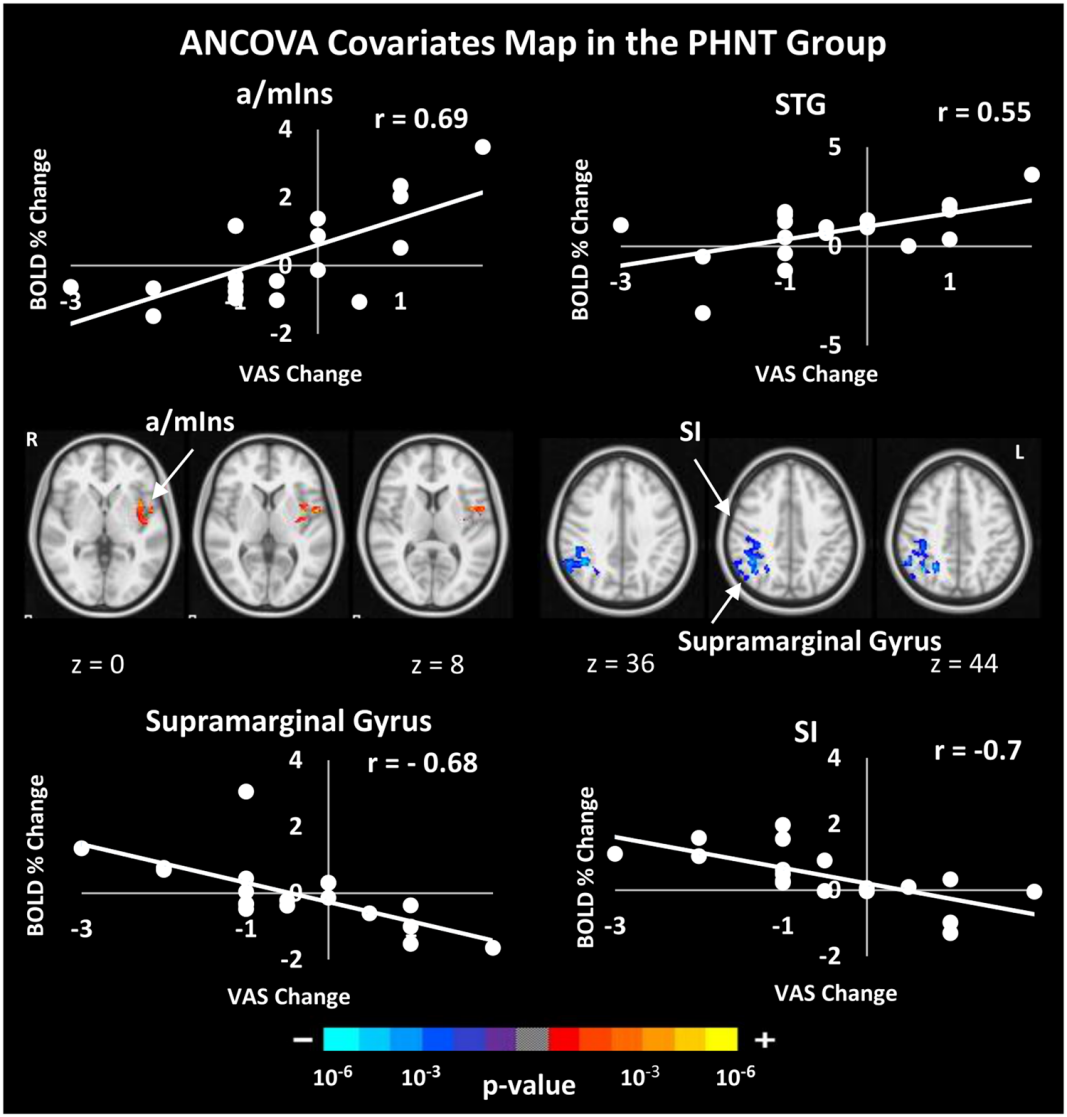
Correlation between subjective brain activity and pain reduction. The PHNT group, but not the REAL, has a significant positive correlation between subjective fMRI signals in the a/mIns and STG and ΔVAS, and significant negative correlation in supramarginal gyrus and SI.

Our event-related fMRI paradigm in conjunction with peripheral autonomic measurements (HR and SCR) gives an opportunity to examine the neural correlates of psychophysiological response induced by acupuncture stimulation. We performed analysis of covariance to investigate the brain correlates of autonomic outflows, belief in acupuncture effectiveness, ΔVAS and MASS index. Interestingly, in the PHNT group but not the REAL group, SCR showed a significant positive correlation with the right dlPFC and the bilateral SI activation (Fig. 9A), and belief in acupuncture effectiveness score showed a significant positive correlation with right dlPFC and vlPFC activation (Fig. 9B). In addition, ΔVAS showed significant positive correlation with left a/mIns and STG and negative correlation with right supramarginal gyrus and SI (Fig. 10). No significant correlation was found between brain data and HR deceleration following REAL or PHNT acupuncture stimulation. MASS index did not show any correlation with subjective brain signal in both groups.

## Discussion

In this study, we used phantom acupuncture, which was devised by our group in a previous study^6,53^ (Fig. 2), in a combined fMRI-ANS experimental paradigm to dissociate acupuncture treatment into needling-specific and non-specific components. Phantom acupuncture is a novel form of sham acupuncture that can induce needling credibility without tactile somatosensory afference. Inducing needling credibility without somatosensory afference is a critical factor for understanding the placebo effect of acupuncture. In our previous study^6^, we demonstrated that the needling specific component (somatosensory needling stimulation) induces sympathetic activation, whereas the needling non-specific components (needling credibility by ritual/contextual influence) result in increased parasympathetic activation, such as decreased heart rate (a shift toward cardiovagal activation) and decreased pupil size, as well as decreased skin conductance response (sympathetic inhibition) after acupuncture needling. Moreover, many different acupuncture sensations were reported by the sham-credible PHNT group, even though those participants received no somatosensory needling stimulation.

### Posterior INS and ACC: Somatosensory Afference-specific Brain Responses

To investigate brain responses to acupuncture somatosensory stimulation, we compared the REAL and PHNT groups. Both REAL and PHNT were credible acupuncture interventions and included visual feedback, though PHNT did not include the component of somatosensory afference. The somatosensory needling stimulation produced by the penetration and rotation of acupuncture needles in the skin has here been considered as the acupuncture needling–specific component^1^. Our results revealed significantly greater acupuncture sensations in the REAL group than in the PHNT group; those sensations were induced from bottom-up sensory processing of needling stimulation via ascending afferent pathways^54^. In the brain, the pIns was activated by the somatosensory needling/afference stimulation in the REAL condition (Fig. 7). A. D. Craig postulated that the anterior and mid insula are involved in the anticipation of pain, whereas somatosensory afference or the actual perception of pain engages the pIns^55–57^, and he further suggested the existence of an ascending sensory pathway that terminates in the pIns^55^. Moreover, the activation of the dorsal posterior insula by temperature, pain (thermal and chronic^58,59^), and different interoceptive modalities (e.g., graded cooling, graded itch, c-fiber touch^60–62^) has been verified using different imaging modalities (see^56^ for review). Connections between the pIns and other subcortical structures, including the basal ganglia and thalamus, might support our evidence of its direct engagement with afferent somatosensory inputs from acupuncture needling stimulation^56,62,63^. Moreover, the pIns receives direct input from slow-conducting unmyelinated C afferents^62^ and afferent projections from the lamina I spinothalamocortical system (a homeostatic afferent pathway that transfer signals from all body tissues) carrying thermal, nociceptive, and other interoceptive signals^56^. Our findings are consistent with those from a recent fMRI study reporting the involvement of pIns in genuine acupuncture stimulation^7^.

Another brain activation unique to real acupuncture stimulation revealed by our results is the ACC. This area is putatively involved in pain anticipation, affective pain evaluation/processing, and endogenous anti-nociception^64^. Moreover, the dorsal sub-region of the ACC (dACC, BA 24) is related to acutely painful stimuli^65^. Acupuncture somatosensory afference can be delivered through an ascending pathway, which carries stimulus information from the spinal cord to the thalamus, PAG, and reticular formation and after that to the ACC, insula, and SI/SII, where tactile information can have broader affective/cognitive influence^66^. The contrast map between STIM and HAND events in REAL further supports the association between the pIns/ACC activation and acupuncture needling.

Thus, the greater activation of the pIns and ACC in response to REAL but not PHNT might be associated with afferent somatosensory inputs and processing the painful stimuli caused by the real acupuncture needling stimulation, respectively, which were absent from the PHNT stimulation group.

### SI and SII: Vicarious Brain Responses to Needling Credibility

Interestingly, our results revealed activation in SI and SII in both the REAL and PHNT groups (Fig. 6). In the REAL group, such activation is a reasonable response to real acupuncture needling; however, in the PHNT group, activation of SI and SII can be explained by two broad interpretations.

First, the visual stimulation carries information about sensory stimulation to participants’ bodies, which could result in SI and SII activation. Indeed, as argued by Posner *et al*., cross-modal links exist between touch and vision in the first stages of sensory processing^67^. The SI is known to be linked to the mirror neuron system, including the posterior parietal and premotor cortices^68,69^. Furthermore, several studies have reported SI activation during the observation of touch^13,70^. For instance, in a 7-T fMRI study, Kuehn *et al*. reported SI activation while their participants observed hand touch^71^. Keysers *et al*. investigated whether an observer’s somatosensory cortices could be activated by watching movies depicting different kinds of touch and found clear SII activation both when participants experienced touch sensations and when they observed someone else getting touched^14,72^. In addition, using a retrograde tracer technique in macaque monkeys, Cipolloni *et al*. studied the cytoarchitecture of the frontoparietal operculum, which is the functional homolog of the SII in humans. Their results showed that the SII receives somatosensory information from the SI, visual information from the extrastriate visual area, and polysensory information from the posterior parietal lobe, which supports the idea that the SII mainly integrates both somatosensory information and information originated from other sensory areas^73,74^. Thus, the SI and SII respond to both experiences of touching and the mere observation of touch. Further evidence that the SII can receive non-tactile input comes from studies about the neural processing of the anticipation of sensory stimulation. For instance, Carlsson *et al*. showed SII involvement in the expectation of touch^75^. In their study, participants were instructed to look at a visual stimulus (either a green or red square) on a screen. As long as the square was green, they would not receive any touch stimulation, but they would be touched on their right foot if the square turned red. Even in the absence of touch, the authors observed SII activation caused by mere anticipation of somatosensory stimulus presented by visual information. Taken together, the SI and SII activation in the PHNT group can be explained either by the observation of the needling stimulation in the video clip and enhanced by needling credibility or by the anticipation of touch from approaching the acupuncturist’s hand as observed in the video clip and supported by the acupuncturist’s presence beside them. This interpretation is supported by the activation of other mirror system regions, as revealed by the conjunction map between the REAL and PHNT groups, such as in the premotor cortex, STS and TPJ, and parts of the intraparietal sulcus (IPS). The IPS contains bimodal neurons that respond to both tactile and visual stimuli^76,77^, which explain its involvement in touch observation. Grefkes *et al*. reported the possibility of IPS activation from visual stimuli alone^78^. These findings are consistent with previous studies in which the mere observation of touch activated some brain regions, including the bilateral SI and SII, premotor cortex, and STS at the TPJ^9,13,79–83^.

Second, SI and SII activation in the PHNT group can be explained by the mirror-touch synesthesia (MTS) phenomenon, in which synesthetes report sensation from the mere observation of someone else’s body part being touched. It is important to note that, although patients in our PHNT-credible group are not synesthetes, they reported acupuncture sensations (e.g., aching and heaviness) associated with the *de-qi* sensation^39^ even without somatosensory needling. Hence, needling credibility leads to a mental rationalization of a perception anticipated by real needling (as all patients were told they will receive real acupuncture stimulation during the acupuncture run) and this also enhanced by the visual afference (video-clip of the needling insertion and stimulation). In fact, our PHNT procedure and MTS have one aspect in common: subjects in both have reported vicarious sensory experiences in the absence of any touch or somatosensory afference. Two theoretical explanations have been presented for the vicarious sensations of MTS: threshold theory, which suggests hyper-activity of the touch mirror system (specifically SI and SII), and self-other theory, which suggests an inability to distinguish the self from others^84–86^. The latter theory is unlikely to explain the acupuncture sensations reported after PHNT stimulation because subjects in the PHNT group found the procedure credible and believed that the stimulation in the video clip happened to their own body. On the other hand, given the significant activation of the SI and SII during PHNT, threshold theory might explain those sensations. Blakemore *et al*. contrasted the observed touch to an object versus to the face in a normal group and in a case ‘C’, who experiences conscious touch by merely observing touch to another person. They found hyperactivity in the sensory mirror system (SI and SII) in C compared to normal controls^13^. This hyperactivity was interpreted as the reason for C’s conscious tactile experiences. In our setting, beyond needling credibility, another contributing factor to the vicarious sensations and SI/SII activity could be that our patients were eager for healing and believed in acupuncture effectiveness. In a multi-modal study of different placebo interventions, Beissner *et al*. have noted robust somatic sensations induced by placebo interventions and interestingly these sanitations reached considerable intensity and extent despite the lack of a peripheral stimulus, and the authors proposed a central etiology for this phenomenon^87^. Although phantom acupuncture evoke somatic sensations in our study, similar sensations has been evoked in previous placebo acupuncture studies^88,89^, so called touch healing^90^, and low-level laser stimulation^91,92^. The evoked sensations in touch healing are called “enhanced touch sensations”^90^. Given that both placebo and real stimulation can elicit somatic sensations that has been associated with clinical efficacy^87,90^, it seems plausible to consider that these sensations have a key role in boosting expectancy and increase patients’ belief about the intervention happening in their periphery and therefore trigger a larger clinical placebo response. In other words, these sensation may be interpreted as a “placebo enhancer”, as early proposed by Beissner *et al*.^87^. Finally, the significant SI/SII activation found in the contrast map between PHNT stimulations and HAND events (Fig. 8B) further supports this interpretation.

Taken together, these findings support the explanation that the significant SI and SII activation and vicarious sensations were induced by observing the acupuncture needling stimulation and enhanced by needling credibility and eagerness for healing. It should be noted that in our previous study^6^ (same paradigm but outside the MRI environment), the reported acupuncture sensations were much greater in amplitude than those reported in this experiment. This difference might result from the distraction and noise of the MRI scanning environment, which distracted participants from focusing on their body sensations. Furthermore, our findings that PHNT reported vicarious sensation and showed pain reduction after phantom acupuncture provides an indirect support for the link between the clinical placebo-effects and placebo-induced sensations.

### dlPFC/vlPFC: Placebo Analgesic Brain Responses

The dlPFC (BA 46) and vlPFC (BA 44, 45) were significantly activated in the PHNT acupuncture stimulation, but not in the REAL stimulation. Consistent activation of these regions were also found in the difference map between PHNT stimulation and HAND events. Interestingly, the right dlPFC and vlPFC showed a positive correlation with the belief in acupuncture effectiveness and expectation scores (Fig. 9). The dlPFC is well known to have a wide range of cognitive functions, including working memory, placebo effect, and anticipation^19,93,94^. Moreover, the dlPFC is known to be associated with cognitive top-down processes for pain control^19,20,95^, as well as the generation, manipulation, and maintenance of cognitive representations, which is consistent with its functions in expectation^31,96^. In a heat-pain paradigm, Krummenacher *et al*. showed that expectation-related placebo analgesia is completely blocked by transient disruption of the left and right dlPFC via repetitive transcranial magnetic stimulation (rTMS). Indeed, inhibition of the dlPFC completely blocked the placebo response, and the authors hypothesized that those results were caused by the disruption of cognitive representations of pain analgesia^96^.

The vlPFC and dlPFC are closely interconnected^28^, and stimulation of one causes ipsilateral co-activation of the other^97^. It has also been hypothesized that the vlPFC is involved in cognitive pain modulation^28^. Furthermore, the vlPFC and orbitofrontal cortex are involved in aversive predicting of error signals and processing of reward expectations^98^, which have repeatedly been reported to be important in the placebo response. Benedetti *et al*. noted that there is no placebo effect without the prefrontal cortex^99^. In a different study, they investigated the altered susceptibility to placebo effects caused by Alzheimer’s disease by studying Alzheimer’s patients at the initial stage of the disease and 1 year later. They found that a smaller placebo response is predicted by reduced frontal connectivity^23,100^. Also, it is known that the experience of pain can be modulated by cognitive factors, such as beliefs and expectations, which is exactly evident in placebo analgesia^96,101^. Taken together, these findings suggest that prefrontal areas (dlPFC and vlPFC) could mediate expectation-induced placebo analgesia in the PHNT group. In line with our hypothesis, we found a significant decrease in pain intensity rating in the PHNT group, reflecting placebo analgesic effects. These data are further supported by our covariate analysis, which revealed a significant correlation between self-reported belief in acupuncture effectiveness score and subjective fMRI signals from the right dlPFC and vlPFC.

The right hemisphere of the brain is more involved in pain processing than the left hemishpere^29^, consistent with its role in withdrawal-related behaviors and negative emotional perceptions^102^. Even though the neural underpinnings for the lateralization of placebo analgesia have not been directly tested, indirect evidence for asymmetric cortical processing has been found in several neuroimaging studies of acupuncture^26^, patients with irritable bowel syndrome^25^, and depression^103^, and all of it suggests preponderance of the right prefrontal cortex in placebo response modulation. For instance, using rTMS over the right dlPFC, Graff-Guerrero *et al*. found increased tolerance of cold pressure pain for both the left and right hands during the rTMS treatment^29^. In addition, several neuroimaging studies have suggested dominant involvement of the right vlPFC and orbitofrontal in the placebo analgesia process^25,26,104,105^. In our study, covariate analysis revealed a significant correlation between the belief in acupuncture effectiveness score and only the right dlPFC/vlPFC fMRI BOLD signal, adding additional evidence for the preponderance of the right prefrontal areas in the placebo response.

Conversely, even though acupuncture is known to reduce pain, we did not find a significant pain intensity (VAS) decrease after the REAL stimulation. We speculate that this might result from cross-modal inhibition between vision (seeing the body being stimulated) and somatosensation (acupuncture needling stimulation)^106^. This speculation is supported by a well-controlled study of Longo *et al*. who observed that vision of one’s own body (either through a mirror or real body) produced a significant reduction in the perception of intensity and unpleasantness of acute pain generated by an infrared laser and also in the amplitude of N2/P2 complex of the nociceptive laser-evoked potentials^106^. In their study, even a non-informative vision of the body affected the pain perception which suggests vision might induce some inhibitory effects on somatosensation. Another support for the competitive relation between visual and sensory modalities may come from the anatomical connectivity between the processing areas of the somatosensory and visual stimulation^107^. Using retrograde tracer in marmosets, Cappe *et al*. found a projection from the visual areas toward the somatosensory areas^108^. These anatomical connections suggest a bottom-up interaction between the incoming tactile and visual information. Further experiments are needed to confirm this speculation by directly comparing the differential clinical effects of acupuncture needling while seeing the body being stimulated and acupuncture needling alone.

### MT+ and STS: Visual Stimulation-specific Brain Responses

Our conjunction analysis between the REAL and PHNT stimulation groups reveals top-down modulation of visual stimulation and bodily attention toward acupuncture stimulation. Visual stimulation via a video of real-time needling stimulation requires visual motion processing of the acupuncture treatment procedure (hand approach to the acupoints and needle rotation). This was reflected by the activation of known visual motion processing areas, including MT+ and STG^109^ (Fig. 6).

### Autonomic Response to REAL and PHNT Acupuncture Stimulation

We used the advantage of our combined fMRI-ANS paradigm to investigate the brain correlates of different autonomic outflow recordings (HR and SCR) to acupuncture stimulation. Our results showed a decreased HR following acupuncture stimulation in both REAL and PHNT groups. Previous research of ANS response has indicated that stimulation by acupuncture needles can induce decreased HR^8,110–112^, and it have been linked to the context of psychophysiological reflexes such as the orienting response (OR)^8,112^. The main characteristics of the OR are stimulus-associated HR deceleration, pupillary dilation and SCR^6,8,113–115^. The OR was further hypothesized to play a role in priming the subject for future sensory evaluation by enhancing the sensitivity to sensory input^116^. In the present study, REAL and PHNT (when credible) induced notable HR deceleration – a hallmark of the OR.

Our data also revealed SCR increase following both REAL and PHNT (when credible) stimulation, with a significant positive correlation between SCR and SI & right dlPFC activation in the PHNT group. In a recent meta-analysis of the brain regions associated with autonomic function processing, Beissner *et al*. found consistent activation of the SI and right dlPFC as a part of the brain network responsible for sympathetic activity regulation as reflected by skin conductivity metrics^117^. In addition, patients with brain damage in the prefrontal cortex have shown a reduction in anticipatory arousal with deficits in producing electrodermal activities^118,119^. In our previous study, we noted that the PHNT-credible, but not the non-credible group, showed a trending significant SCR increase following stimulation events^6^. Taken together, these findings suggest that r-dlPFC is involved in anticipatory regulation of arousal (reflected by the increased SCR) from acupuncture stimulation, enhanced by credibility of the procedure and belief in acupuncture effectiveness.

In line with these findings, in our other previous study we noted, on average, increased SCR and HR deceleration following real and non-insertive sham acupuncture, though the response magnitude was greater following real acupuncture^8^. The fact that belief in acupuncture effectives showed a positive significant correlation with the increased SCR following phantom acupuncture stimulation, and both of them showed a significant correlation with the subjective fMRI signal in the right dlPFC, might provide a link between expectancy, SCR, right dlPFC activation and the placebo response found in the PHNT group, a hypothesis that need to be directly tested in the future studies.

Several limitations should be noted in this study. First, the sample size was quite small, so that we didn’t have enough patients in the PHNT non-credible group to perform statistical comparisons between the credible and non-credible PHNT groups. Second, the baseline of the pain rating (Pre-VAS) for the REAL group was significantly lower than the PHNT group which made it difficult to compare the behavioral effects of the two kinds of interventions. Third, further experiments are needed to investigate cross-modal inhibition effects between vision and somatosensation, which might explain why the REAL group didn’t report significant pain reduction after the real acupuncture stimulation. Finally, our covariate analysis did not show a correlation between SI/SII activation and the MASS index that would suggest a direct link between the reported vicarious sensations and sensory mirror system activation. This might be due to the narrow dynamic range of the MASS index in PHNT.

In conclusion, we used a form of placebo acupuncture, *phantom acupuncture*, to dissociate the neural correlates of the different components of acupuncture treatment in an event-related fMRI experiment. Our study suggests that phantom acupuncture can be a viable sham control because it reproduces the acupuncture needling procedure without somatosensory tactile stimulation and can be delivered in a credible manner ($\simeq$82% credibility rate). Needling credibility and being eager for healing might have led to the SI/SII activation and vicarious acupuncture sensations reported by the PHNT credible group. The needling stimulation component is associated with brain activation in the pIns and ACC, reflecting needling-specific brain responses. Furthermore, expectation and belief in acupuncture effectiveness could induce expectation-related placebo analgesic effects, leading to a reduction of subjective pain intensity. Our data suggest that the level of brain activity in the prefrontal cortex, particularly in the right hemisphere, defines the contribution of expectations and belief to placebo analgesia.
